# An Indoor Navigation System for the Visually Impaired

**DOI:** 10.3390/s120608236

**Published:** 2012-06-13

**Authors:** Luis A. Guerrero, Francisco Vasquez, Sergio F. Ochoa

**Affiliations:** 1 Computer Science and Informatics School, Universidad de Costa Rica, San José 2060, Costa Rica; E-Mail: luis.guerrero@ecci.ucr.ac.cr; 2 Department of Computer Science, Universidad de Chile, Av. Blanco Encalada 2120, 3er Piso, Santiago 837-0459, Chile; E-Mail: fvasquez@dcc.uchile.cl

**Keywords:** indoor positioning, movement detection, navigation system, augmented object, voice interface, visually impaired

## Abstract

Navigation in indoor environments is highly challenging for the severely visually impaired, particularly in spaces visited for the first time. Several solutions have been proposed to deal with this challenge. Although some of them have shown to be useful in real scenarios, they involve an important deployment effort or use artifacts that are not natural for blind users. This paper presents an indoor navigation system that was designed taking into consideration usability as the quality requirement to be maximized. This solution enables one to identify the position of a person and calculates the velocity and direction of his movements. Using this information, the system determines the user's trajectory, locates possible obstacles in that route, and offers navigation information to the user. The solution has been evaluated using two experimental scenarios. Although the results are still not enough to provide strong conclusions, they indicate that the system is suitable to guide visually impaired people through an unknown built environment.

## 1. Introduction

People with visual disabilities, *i.e.*, partially or totally blind, are often challenged by places that are not designed for their special condition. Examples of these places are bus and train terminals, public offices, hospitals, educational buildings, and shopping malls. Several “everyday” objects that are present in most built environments become real obstacles for blind people, even putting at risk their physical integrity. Simple objects such as chairs, tables and stairs, hinder their movements and can often cause serious accidents.

Several proposals have tried to address this challenge in indoor and outdoor environments [1]. However most of them have limitations, since this challenge involves many issues (e.g., accuracy, coverage, usability and interoperability) that are not easy to address with the current technology. Therefore, this can still be considered an open problem.

This paper presents a prototype of a navigation system that helps the visually impaired to move within indoor environments. The system designed has been focused on usability of the solution, and also on its suitability for deployment in several built areas.

The main objective of the system is to provide, in real-time, useful navigation information that enables a user to make appropriate and timely decisions on which route to follow in an indoor space. In order to provide such information, the system must take into account all the objects in the immediate physical environment which may become potential “obstacles” for blind people. This kind of solution is known as a micro-navigation system [1]. Two main aspects should be addressed by this system to provide navigation support: (1) detection of the position and movement intentions of a user, and (2) positioning of all the objects or possible obstacles into the environment.

**Figure 1.**
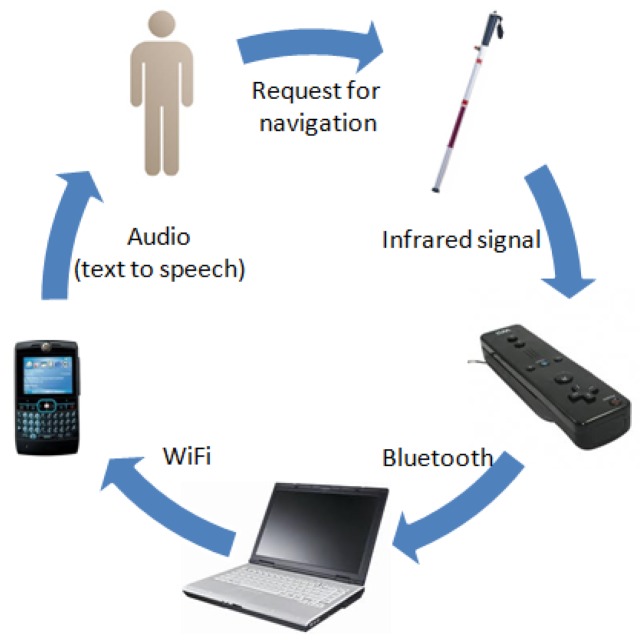
Components of the navigation system.

In order to deal with these issues the solution uses the interaction among several components as a platform to capture and process the user and environment information, and to generate and deliver navigation messages to users while they are moving in an indoor area. The system's main components are the following: an augmented white cane with various embedded infrared lights, two infrared cameras (embedded in a Wiimotes unit), a computer running a software application that coordinates the whole system, and a smartphone that delivers the navigation information to the user through voice messages (Figure 1).

When the users request navigation information, they push a button on the cane. It activates the infrared LEDs embedded in the cane. The software application instantly tries to determine the user's position and the presence of obstacles in the surrounding area. The user's position and movement are detected through the infrared camera embedded in the Wiimotes. These devices transmit that information via Bluetooth to a software application running on the computer. The application then uses such information and also the data that it has about the obstacles in the area, to generate single navigation voice messages that are delivered through the user's smartphone. The system follows a common-sense approach [2] for the message delivery.

The system prototype has been tested in two different scenarios. Although these results are still preliminary, they indicate the proposal is usable and useful to guide the visually impaired in indoor environments. The next section presents and discusses the related work. Section 3 establishes the requirements of the solution. Section 4 describes the navigation process and its main components. Section 5 explains the design and implementation of the software application that is in charge of coordinating all peripherals and processing the system information in order to deliver useful navigation messages to end-users. Section 6 presents and discusses the preliminary results. Section 7 presents the conclusions and future work.

## 2. Related Work

Many interesting proposals have been done in the area of location and mobility assistance for people with visual disabilities. These works are mainly focused on three types of environments [1]: outdoor [3–5], indoor [6–9], and some mix of the previous ones [10–12].

The research on outdoor environments mainly addresses the problem of users positioning during micro-navigation and macro-navigation [1]. Micro-navigation studies the delivery of information from the immediate physical environment, and macro-navigation explores the challenges of dealing with the distant environment. In both cases the use of global positioning systems (GPS) has shown to be quite useful in recognizing the user's position.

The studies focused on indoor environments have proposed several *ad hoc* technologies and strategies to deliver useful information to the user [1]. However, just some of them are suitable to be used by visually impaired people. For example, Sonnenblick [9] implemented a navigation system for indoor environments based on the use of infrared LEDs. Such LEDs must be strategically located in places used by the blind person to perform their activities (e.g., rooms and corridors), thus, acting as guides for them. The signal of these guiding LEDs is captured and interpreted by a special device which transforms it into useful information to support the user's movements. The main limitation of such a solution is the use of an infrared receptor instead of a device with large coverage such as an infrared camera. Because the infrared signal must be captured to identify the user's position, the receptor device must point directly at the light source (e.g., the LEDs), thus losing the possibility of smooth integration between the device and the environment.

Hub, Diepstraten and Ertl [6] developed a system to determine the position of objects and individuals in an indoor environment. That solution involved the use of cameras to detect objects and direction sensors to recognize the direction in which the user is moving. The main limitation of that proposal is the accessibility of the technology used to implement it, since the system requires a specialized device to enable the user to interact with the environment. This system also pre-establishes possible locations for the cameras, which also generates several limitations; for example the detection process requires the person points out his white cane at the eventual obstacles.

In a later work, Hub, Hartter and Ertl [7] went beyond their previous proposal and included in the system, the capability of tracking various types of mobile objects, e.g., people and pets. Then, using an algorithm similar to human perception, they attempted to identify such tracked objects by comparing their color and shape, with a set of known objects.

Treuillet and Royer [11] proposed an interesting vision-based navigation system to guide visually impaired people in indoor and outdoor environments. The positioning system uses a body mounted camera that periodically takes pictures from the environment, and an algorithm to match (in real-time) particular references extracted from the images, with 3D landmarks already stored in the system. This solution has shown very good results to locate people in memorized paths, but it is not suitable to be used in environments that are visited for the first time. The same occurs with the proposal of Gilliéron *et al.* [13].

There are also several research works in the robotic and artificial intelligence fields, which have studied the recognition of indoor scenes in real-time [14–17]. Some of these solutions allow for creating the reference map dynamically, e.g., the vision-based systems proposed by Davison *et al.* [18] and Strasdat *et al.* [19], or the ultrasound positioning system developed by Ran *et al.* [12]. Although they have shown to be accurate and useful in several domains such as robotics, wearable computing and the automotive sector, they require that the vehicle (in our case the blind person) carry a computing device (e.g., a nettop) to sense the environment and to process such information in real-time. Since they must carry the white cane with them all of the time, the use of extra gadgets that are not particularly wearable, typically jeopardizes the suitability of such solutions [20]. In that sense the solution proposed by Hesch and Roumeliotis [21] is particularly interesting because they instrumented a white cane, which is a basic tool for blind people. However, such a solution has two important usability limitations: (1) the sensors mounted in the cane (a laser scanner and a 3-axis gyroscope) are too large and heavy, which limits the user movements, and (2) the laser scanner in the cane is directional, therefore it has the same limitations as the previously discussed infrared-based solutions.

Radio Frequency Identification (RFID) is a technology commonly used to guide the visually impaired in indoor environments. For example, in [22,23] the authors propose a system based on smartphones that allows a blind person to follow a route drawn on the floor. This solution combines a cane with a portable RFID reader attached to it. Using this cane, a user can follow a specific lane, composed by RFID labels, on the floor of a supermarket. Kulyukin *et al.* [24] propose a similar solution, replacing the white cane with a robot that is in charge of guiding the visually impaired person.

Another RFID-based solution that supports the navigation of visually impaired people was proposed by Na [25]. The system, named BIGS (for Blind Interactive Guide System), consists of a PDA, a RFID reader, and several tags deployed in the floor. Using these elements the system can recognize the current location of the PDA's user, calculate the direction of the user, recognize voice commands and deliver voice messages. The main limitations of using RFID-based solutions are two: (1) the reconfiguration of the positioning area (e.g., because it has now a new setting) involves more time and effort than other solutions, like vision-based systems, and (2) the users are typically guided just through predefined paths.

An important number of works have also been conducted to determine users' position and perform object tracking in indoor environments as support of ubiquitous computing systems [26–29]. Although they have shown to be useful to estimate the users' position, they do not address the problem of obstacles recognition in real-time systems. Therefore, they are partially useful in addressing the stated problem.

Hervás *et al.* [30] introduced the concept of “tagging-context” for distributing awareness information about the environment and for providing automatic services to the augmented objects used in these environments. Finally, an interesting work regarding a network of software agents with visual sensors is presented in [31]. This system uses several cameras for recognition of users and tracking of their positions in an indoor space. However these last two works do not address the particularities involved in the supporting of such navigation for the visually impaired.

## 3. Requirements of the Solution

A list of functional and non-functional requirements were defined for the proposed navigation system. Such requirements were based on the results of the study conducted by Wu *et al.* [32] and also on the experience of the authors as developers of solutions for blind people. These requirements were then validated with real end-users. Some non-functional requirements, such as privacy, security and interoperability, were not considered in the system at this stage, as a way to ease its evaluation process.

The general requirements that were included in the design of the proposed micro-navigation system were classified in the following categories:

1. *Navigation model*. These requirements are related to the model and not to its implementation. The following are main requirements related to such a component:
  - *Generality.* The navigation model must be able to be used (with or without self-adaptations) in several indoor environments. It ensures that the user will count on navigation support in an important number of built areas.
  - *Usefulness*. The model must allow the detection and positioning of mobile users and obstacles, and based on that, it has to provide useful navigation information.
  - *Accuracy.* The model must count on quite accurate information about the user's movement and location. The information accuracy must allow the system to support the navigation of blind people in a safe way. Based on the authors' experience, the navigation system should be suitable if the worst case has a positioning error of 0.4 m. In the worst case the system must detect the user's movement in 500 ms.
  - *Feasibility*. The model implementation should be feasible using technologies that are accessible (in terms of cost, usability and availability) to the end-users.
  - *Multi-user*. The model must consider the presence of more than one user in the same environment.
2. *Navigation system*. The requirements related to the implementation of the navigation model, *i.e.*, navigation system, are the following:
  - *User-centric.* The services provided by the system must consider the particular disabilities of the users, e.g., their level of blindness.
  - *Availability:* The system must maximize the availability of its services independently of the environment where the user is located. Thus, the solution becomes usable in several built areas.
  - *Identification.* The system must be able to unequivocally identify the users.
  - *Multi-user.* The navigation services provided by the system must consider the eventual participation of more than one user in the same environment.
  - *Performance:* The system performance must be good enough to provide navigation services on-time, considering the users' movements, the walking speed and the obstacles in the environment. Based on the authors' experience, the response time to end-users was set in 300 ms.
  - *Usability.* The use of the system should be as natural as possible for the end-users.
  - *Usefulness.* The information delivered by the system must be useful and allow the users to navigate indoor environments properly, even if they are visiting those spaces for the first time.
  - *Economical feasibility.* The cost of the system must be affordable to the end-users.

## 4. Navigation Process

**Figure 2.**
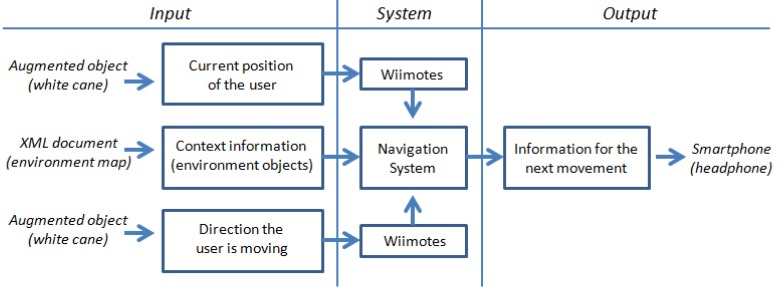
Main structure of the navigation process.

The navigation process uses three key pieces of information to analyze the current situation and deliver useful navigation information to the user (Figure 2). These pieces of information are: (1) the user's current position in the environment, (2) the direction in which the user is moving, and (3) the presence of objects in the surrounding area that may be potential obstacles. We next explain how this information is captured and delivered by the system components introduced in Figure 1.

### 4.1. Detecting Users Position and Movements

**Figure 3.**
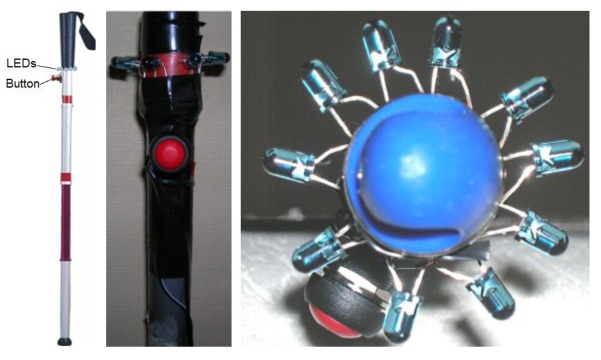
The augmented white cane.

The white cane that helps determine the user position and movement is an augmented object [33], which includes several infrared LEDs, and a button that allows the user to activate and deactivate the navigation system (Figure 3). The cane was chosen as the most suitable object to be augmented and thus represent the user position, after applying the Augmented Objects Development Process (AODeP) methodology [34] to a list of possible candidate objects.

The application of such a methodology also allows us to identify the following key requirements to be accomplished by the object to be augmented: (1) it must be able to emit infrared light that can be captured by the Wiimote; (2) it must be located in (or worn by) the user, so that it moves with the user; (3) it must be small and light enough to not hinder the movement of the user; and (4) it should minimize the cognitive effort required to use it. After analyzing several alternatives the white cane was identified as the most appropriate object to indicate the user position, considering not only the stated requirements, but also the object availability and the navigation system accuracy.

The infrared LEDs were installed in a radial form to ensure visibility between the light source and receptor (*i.e.*, the Wiimote). This allows the system to receive the data of the light source location in a consistent manner. It is an ideal situation to deliver accurate information on-time to the user. The batteries were embedded in the actual stick so that they would pass unnoticed. A small button turns the LEDs on and off. Thus, the user is always in control of the system all the time.

**Figure 4.**
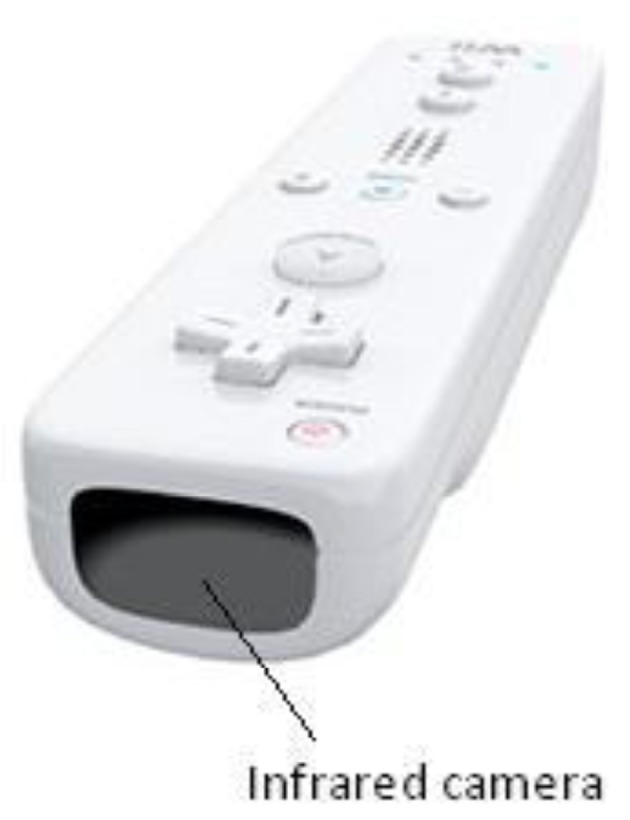
Regular Wiimote.

Regular Wiimotes were used to detect the users' position in the indoor environment. These devices act as the movement and location sensors of the environment. Although these controls have several useful capabilities, only its infrared camera was used in this proposal. This camera is located in the front of the control (Figure 4) and it is able to detect movements of infrared lights (e.g., those installed in the augmented white cane) to no more than 10 m.

The Wiimote calculates the relative position of the light source. Therefore, by setting up the Wiimote in an indoor space, and knowing the exact position of the control, we can identify the position of any other object emitting infrared light in that area.

Once the white cane position is recognized by the Wiimote, it transmits this information to a computer that is in charge of processing it and to deliver the corresponding messages to the user's smartphone. The whole process must be done quickly, since the user that has to feel the navigation system responds in real-time.

The technologies involved in this solution allow reaching such a goal. For example the Wiimote has a dedicated microchip to control the Bluetooth antenna, which transmits to 2.1 Mbits/s. Therefore, a Wiimote can perform fast interactions with the computer controlling the whole system.

Concerning the WiFi network, the computer uses a Mobile Ad hoc Network (MANET) [35] to interact with the smartphone. All interactions between devices were considered to one-hop, due the communication threshold of WiFi networks deployed in build area is at least 10 m in almost any case. That threshold would be enough, because 10 m is also the maximum communication range considered in this work for Bluetooth communication links. These restrictions make this proposal usable in rooms of no more than 30 m^2^. That area can be extended considerably if we use WiFi instead of Bluetooth, but at this moment it is not particularly relevant due the proposal is in a preliminary evaluation phase.

The interactions performance on a MANET could be a problem, because that network type usually loses data packets. Devices interacting to one-hop in the MANET can expect a reference throughput of 800–1,000 Mbits/s [35]. Although this is enough to perform fast interactions, several MANET implementations are not able to achieve such a transfer rate, therefore it is important to evaluate the WiFi link to be sure that is able to count on an appropriate performance.

### 4.2. Managing the Environment Information

In order to carry out the positioning process, the system requires obtaining some initial parameters related to the indoor environment and the Wiimotes' position. These parameters allow the system not only to estimate the users' position, but also to understand the data obtained from the controls.

**Figure 5.**
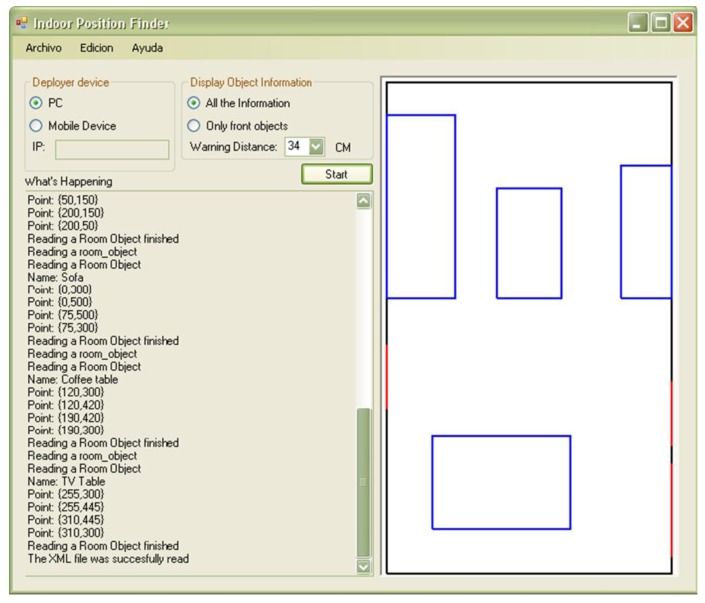
Application to describe indoor environments.

The parameters are retrieved from an XML file that the software application (running on the laptop) keeps for such purposes. The file contains the information describing the room and the objects contained on it, including obstacles and the Wiimote coordinates. In order to avoid that developers have to edit and update the XML file when performing the setup process, we have developed a software application that allows them to manage such information in a visual way (Figure 5).

The developer defines the borders of the environment and draws all the objects in it. Based on that, the system generates the XML specification of the setting information. The format used to represent the information allows the system to describe indoor environments in an understandable and structured way. This facilitates the information management and interoperability.

**Figure 6.**
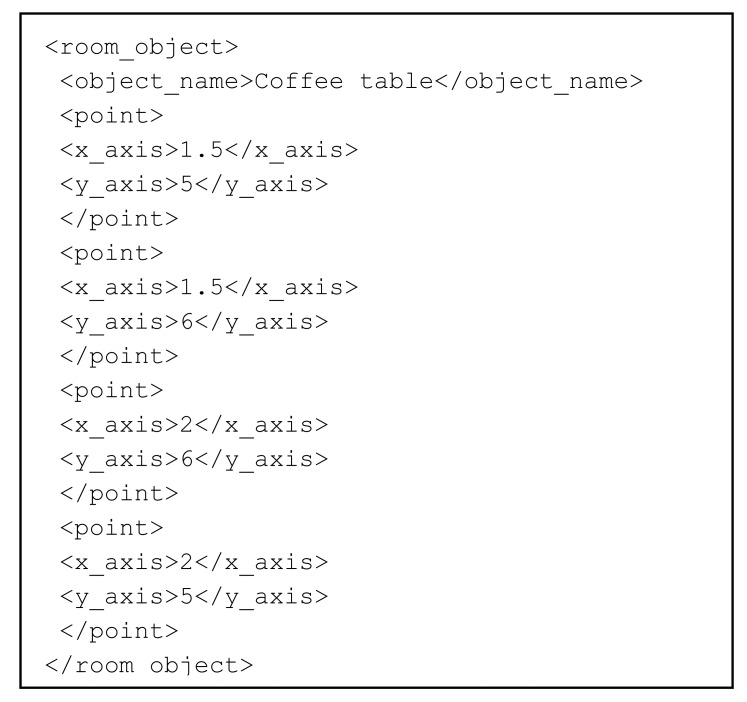
XML file containing information about objects in the room.

Some of the data elements required in the XML file are the following: room length and width, relative position of each Wiimote, angle in which the controls were fixed to the walls, and the relative position of each object (*i.e.*, possible obstacles) in the room. Figure 6 shows part of a XML file, which corresponds to the definition of an object named “coffee table”. This example shows a tag labeled as < room_object>, which includes several features describing the object. The object definition includes the object name, specified using the < object_name> tags. This name is the one the system is going to indicate to the user when it delivers navigation information that involves such an object. The object definition uses the < point> tag to identify the object corners. All coordinate are represented in meters.

The process of establishing the points of an object follows a predefined heuristic. The first point to be referred is the lower left point of the object. The order to refer the rest of the points of an object is established by bordering the object clockwise. This heuristic, used to refer the points in a room, must be utilized consistently when specifying objects contained in an indoor setting, as it adds extra semantics to the stored information.

**Figure 7.**
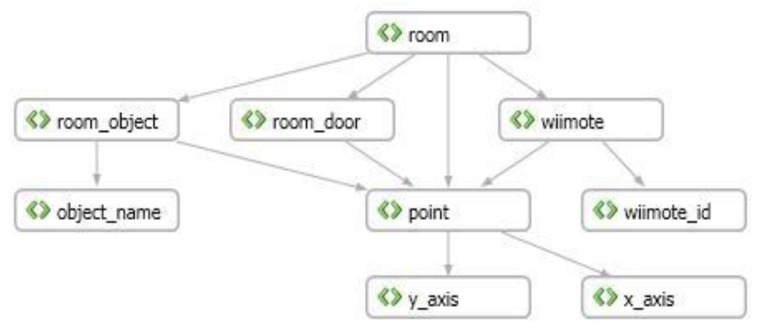
Tree structure representing the XML file that describes a room.

Analyzing this XML file we can generate a complete map of the room, recognizing the environment in which the user is located. Figure 7 shows an example of a map generated from a XML file. To facilitate the comprehension of the map, it is represented as a tree.

This map presents only the possible relationships between the objects specified in the XML file, but it does not indicate the number of instances of those objects that are included in each component of the structure. For example, in the case of an object in the room (< room_object>), its representation includes at least four points (< point>) as indicated in the previous example. However such cardinality is not represented in the structure presented in Figure 7.

### 4.3. Recognizing the User's Position

In order to understand the positioning strategy used by the proposed solution, it is important to comprehend how the Wiimote controls capture the user's position. The relative position of the infrared emitter is established in a plane that is perpendicular to the direction in which the infrared camera points. However, this information is not sufficient to determine the user's relative position in the room. Therefore, a second Wiimote is required to establish the relative position of the infrared source with respect to this second sensor. Considering these two views, it is possible to determine the location of the light emitter. Then, through a triangulation process, we can estimate the relative position of the user in the room.

**Figure 8.**
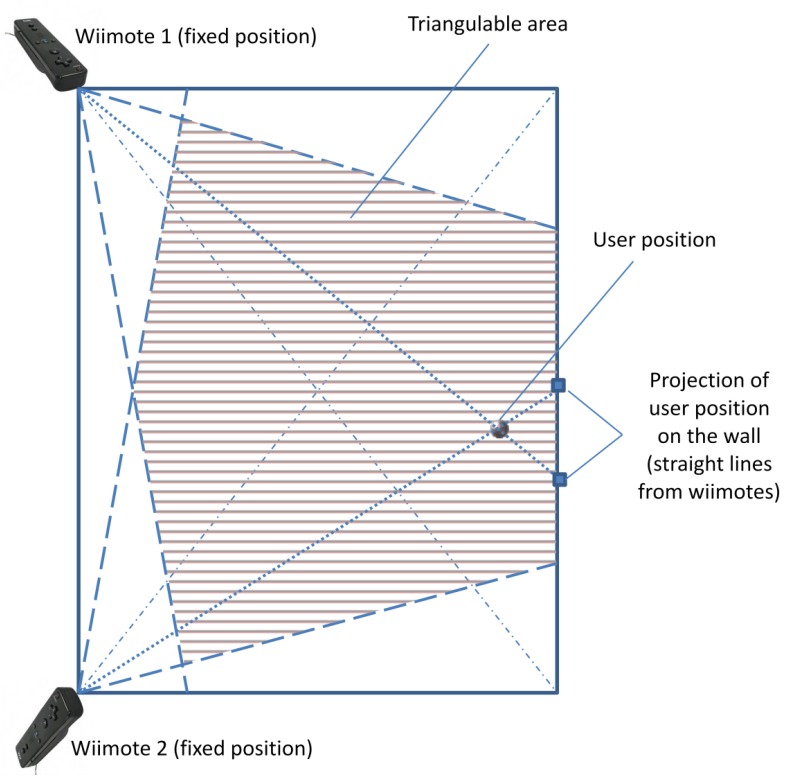
Elements participating in the triangulation process.

In this case, the triangulation process uses the information captured by every Wiimote and generates two straight lines (Figure 8). These lines must connect the point at which the Wiimote is located and the point identifying the user's location. The first point is known as it is part of the initial settings and therefore such information is stored in the XML file.

The second point is *a priori* unknown. However, the Wiimote can notify the angle generated by this line, and thus calculate the projection of such a line on the opposite wall of the room. Using this second point, for which we already have data to calculate it, it is possible to determine the equation of the associated straight line. Since this operation is done for both Wiimotes, we can calculate the intersection of the two lines which corresponds to the exact position of the user.

This simple strategy to calculate the user's position based on triangulation works well for rectangular rooms. However, *a priori* it is not so clear if this strategy is applicable to other room shapes. If we consider that the walls of the room do not need to be “real”, we can generalize this solution to other shapes of room.

As part of a more general solution, the system is able to create “virtual walls”, which correspond to the sides of the square bounding the room. Thus, it is possible to project the straight line on the sides of such a square obtaining the same results explained above. This capability makes the solution suitable for use in several built areas, regardless the shape of the room in which the user is located. Although this projection overcomes the limits of the room, the intersection of the two straight lines should always be inside the positioning area.

In this proposal, the position of the origin point must correspond to the lower left corner of the square bounding the room. This convention allows us to simplify the calculations made by the positioning algorithm. This restriction depends only on the way in which the person looks at the room, therefore, almost any corner of the specified square may correspond to the lower left corner. Independent of the origin point, the convention established for the definition of all points representing the room (described in the XML file), must be respected. This allows us to model any room in a similar way, describing the walls as objects in the room.

A limitation we have identified in this process is present when the room is exactly a square. In that case, the algorithm requires that the Wiimotes not be located at the opposite corners of the room and at the same angle with regard to the walls. If that were the case, the algorithm would not be able to triangulate the user position, since both controls are located in symmetric places. Thus, both Wiimotes look at the same level, and therefore deliver the same information to the software component running in the laptop. This information is insufficient to calculate the position of the user.

In order to deal with this restriction, we can locate the controls in a position so that they share a wall. The same effect is generated if we locate the Wiimotes in different angles with respect to the walls, although they remain on opposite corners of the room. Thus, the symmetry of the view related to every Wiimote is broken and therefore the triangulation can be done following the regular process.

The equations used to calculate the straight lines and perform the triangulation process are shown in Equation (1). This simple system of equations describes the general case for triangulating two straight lines in a two-dimensional plane. In our case, the indoor environment is represented as a two-dimensional plane, where the origin point (*i.e.*, X = 0, Y = 0) always corresponds to the lower left corner (*i.e.*, Wiimote 2 in Figure 8):

(1) $$\begin{array}{l} \begin{array}{c} Y-Y_{w1}=m_{1}\left(X-X_{w1}\right)\\ Y-Y_{w2}=m_{2}\left(X-X_{w2}\right) \end{array} \end{array}$$

where:

$$m_{i}=\frac{Y_{pi}-Y_{wi}}{X_{pi}-X_{wi}};$$

(*X_pi_,Y_pi_*) ≔ *Projection of the straight line related to the Wiimote i* (see Figure 8);

$$\left(X_{wi},Y_{wi}\right):=\text{Position of the Wiimote i}$$

According to the system of equations shown in Equation (1), for obtaining the exact position (X, Y) of the user in a 2D plane, we can use the equations shown in Equation (2). The preliminary tests done to the system indicate the error range is 10–15 cm. In these tests we analyzed the error range in two different rooms to mitigate the effect produced by using a single Wiimotes distribution. Every test (one per room) considered the detection of three obstacles from three different angles. During the tests the user walked towards an obstacle using different routes. At the moment that a warning message is given to him, the user position indicated by the system was compared with the real position. The maximum error identified was 15 cm, but in most cases it was below 10 cm.

(2) $$\begin{array}{c} X=\left[\frac{\left(m_{2}X_{w2}-m_{i}X_{w1}\right)-\left(Y_{w2}-Y_{w1}\right)}{\left(m_{w2}-m_{1}\right)}\right]\\ Y=m_{2}\left[\frac{\left(m_{2}X_{w2}-m_{1}X_{w1}\right)-\left(Y_{w2}-Y_{w1}\right)}{\left(m_{2}-m_{1}\right)}\right]-m_{2}X_{w2}+Y_{w2} \end{array}$$

By calculating the user's position iteratively, it is possible to identify the direction in which the person is moving in the environment. The preliminary tests indicate the system detects the user's movement almost in real time; however that time does not have been measured formally. Then, using the information related to the direction in which the person is moving and his current position, it is possible to determine if he is approaching some potential obstacle specified in the XML file. This also allows the system to inform the user about the position of all obstacles in the room. For example, the system could inform the user of the existence of an object nearby when the user is moving towards it. The system could also describe all the objects that are around the user or those located within certain a distance from him. Moreover, it can indicate the angle at which the objects are, given the direction in which the user is moving. This orientation paradigm is well-known to blind people and it is known as “the clock paradigm”. The following section describes the navigation module, which is a central piece of this solution.

## 5. The Navigation Module

**Figure 9.**
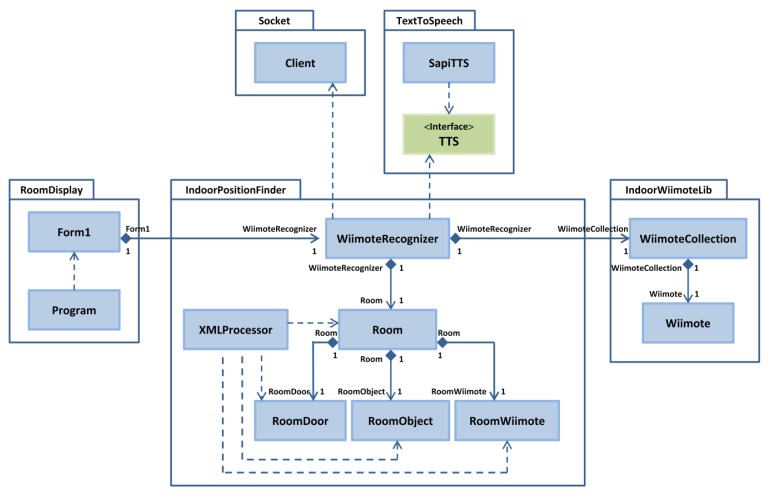
Structure of the navigation module.

This software module that runs on the laptop (or in any other similar computing device, e.g., a netbook, a nettop) acts as the coordinator of the whole system. The coordination activities involve the use of five components (Figure 9): *IndoorPositionFinder*, *WiimoteLib*, *RoomDisplay*, *TextToSpeech*, *Socket*. Next, we briefly describe each of them:

### 

#### IndoorPositionFinde

This component is central to the navigation module since it stores most of the system logic used for positioning and tracking objects. This component consists of six sub-components: *RoomWiimote* (manages the information coming from the Wiimotes registered in the room), *RoomObject* (manages the information about the obstacles in the room), *RoomDoor* (manages information about doors into the physical environment), *Room* (represents a room, and contains the collections of objects present in the room), *XmlProcessor* (interprets the XML file representing the room and its objects) and *WiimoteRecognizer* (calculates positions, distances, and movement directions of a user). The *IndoorPositionFinder* component also generates navigation messages to be sent to the end-user.

#### WiimoteLib

This public library (http://www.codeplex.com/WiimoteLib) [36] contains the services required to interact with the Wiimotes and understand the information they provide to the application. In our case, just some small modifications were done to the services provided by the library, in order to adapt them to the performance needs imposed by our application scenario. Although such a library provides many services, this application uses just two of them: *Wiimote* (connects the system to a Wiimote) and *WiimoteCollection* (similar to the previous one, but the connection is performed by a set of Wiimotes).

#### RoomDisplay

This component allows administrating the system through a graphical user interface (GUI). It is composed of two sub-components: *Form1* (represents the GUI) and *Program* (opens the sub-component *Form1*).

#### TextToSpeech

This component allows delivery of an audio message to the user. It is composed of the following sub-components: *TTS* (an interface that establishes the structure of a text-to-speech class), and *SapiTTS* (implements the TTS interface using Microsoft Text-To-Speech library).

#### Socket

This component creates and manages a socket (*i.e.*, a communication channel) between the computer and the user's smartphone, when navigation messages must be delivered to the user. This component involves two sub-components: *Client* (generates a socket client and delivers an audio stream containing the navigation information for the user), and *Server* (generates a server process that receives an audio stream and reproduces it).

The navigation module, particularly the *IndoorPositionFinder* component, keeps running at all times and waits for a request of navigation supporting information. When the system detects an infrared light, it assumes the user is asking for help and therefore it triggers a thread to address the request. The process performed by this component to provide such information is the one described in Section 4 (see Figure 2).

## 6. Preliminary Results

In Sections 6.1 and 6.2 we report on the experiments performed using the proposed system. Moreover, in Section 6.3 we present some metrics on non-functional requirements of this solution. Section 6.4 discusses the preliminary results.

### 6.1. First Evaluation Process

**Figure 10.**
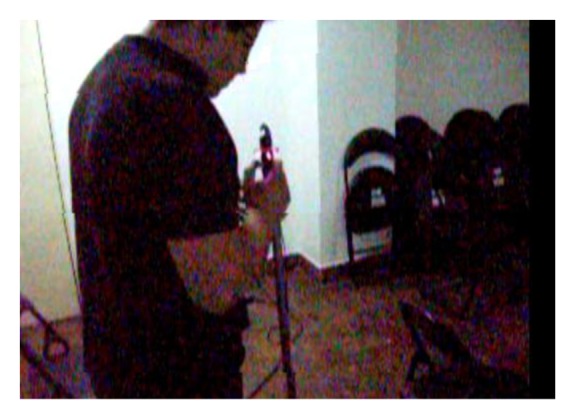
Use of the augmented cane during the evaluation process.

During the first experimentation process, it was not possible to count on visually impaired people to perform the test. Therefore, we decided to begin a pre-experimentation process with blindfolded people in order to run a first analysis of the proposed solution (Figure 10). Five volunteers participated in this experiment.

In order to make the participation interesting for these volunteers, we set up the following challenge: they should all be able to cross a room just by using the information delivered through the application. The type of information delivered to them does not intend to indicate which movements the user must perform. It only provided contextual information that helped them make their own decisions about which route to follow. For example, if the person is moving towards a table, the system does not assume that the user must dodge it, since the user could be intentionally going to the table to pick up something. Therefore, the system informs the user if moving towards an object or a possible obstacle, and what type of object it is. For example “An object is one meter ahead”. These messages are configurable; therefore they can be changed according to the user's preferences.

As shown in Figure 5, the warning distance is a configurable parameter. In this test the warning distance was set to 1 m. Other parameter that can be established according to the user's preferences is the waiting time between messages, *i.e.*, the minimum time the system waits before delivering a next message to the user. In this experimentation such a time was set to 2 s.

**Figure 11.**
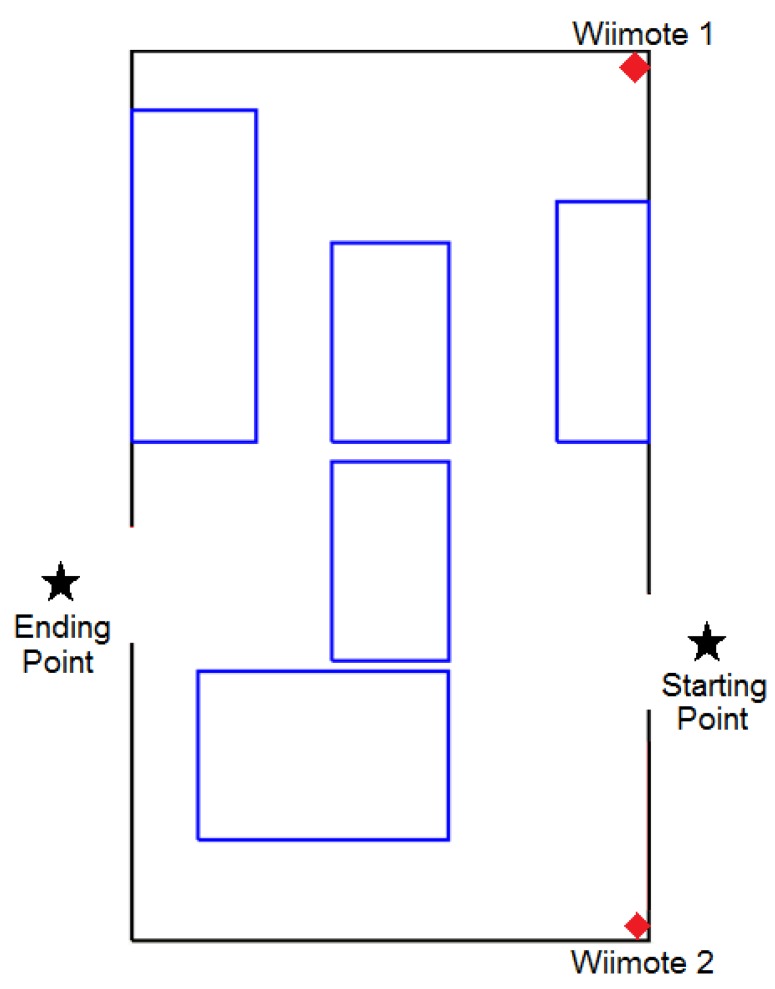
Distribution “A” of the experimentation room.

The room used in this experiment was 20 m^2^ approximately and it consisted of several obstacles which made the test more interesting (Figure 11). Two versions of the same system were used by each participant and a score was obtained in each test. One version of the system delivered the audio messages through the speakers installed in the room, and the second one used the speakers of the smartphone.

The distribution of obstacles in the room was changed after each test so that the users would not memorize the room setting. Two equivalent object distributions were used in this experiment. The users randomly selected the room distribution and the version of the system to be used first.

The obtained results were divided into two groups: *with* and *without a smartphone*. In both cases, the volunteers were asked five questions to capture their impressions regarding the system. The responses were obtained as soon as the participants finished the test to get their perception of the experience. Moreover, each participant performed the tests using both versions of the system in a consecutive way. We then tried to capture comparative scores between these two alternatives of the system.

Participants had to use a scale of 1 to 10 to represent their responses. For this scale it was considered that the distance between any pair of adjacent numbers is exactly the same (for example, the distance between 1 and 2 is exactly the same as from 2 to 3, and so on). The questions made to the participants were the following:

1. On a scale of 1 to 10, where 1 is “hard to use” and 10 corresponds to “easy to use”, how do you rate the system usability?
2. On a scale of 1 to 10, where 1 is “incomprehensible” and 10 is “clear”, how understandable was the information delivered by the system?
3. On a scale of 1 to 10, where 1 corresponds to “useless” and 10 corresponds to “very useful”, how useful is the information provided by the system?
4. On a scale of 1 to 10, where 1 corresponds to “very delayed” and 10 corresponds to “immediate”, how fast is the delivery of information from the system?
5. On a scale of 1 to 10, where 1 is “very imprecise” and 10 corresponds to “exact”, how precise was the contextual information provided by the system?

**Figure 12.**
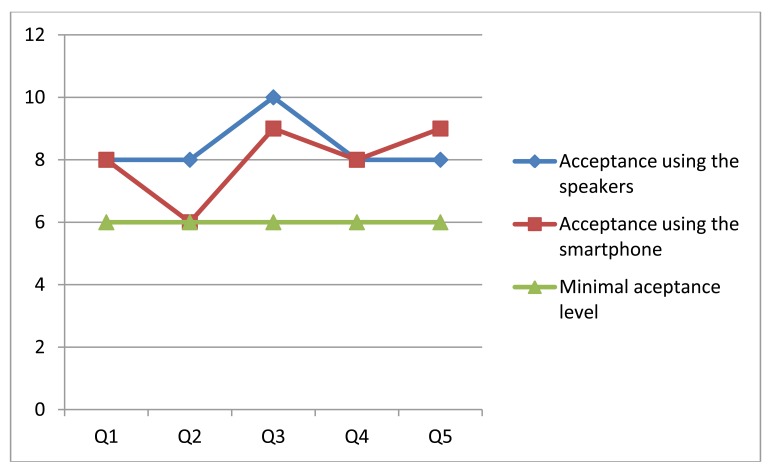
Median of the experimental results with and without a smartphone.

Given the questions and weights indicated by the users, a general criterion of acceptability was defined for each evaluated item. In order to do that, the tests and the obtained results were analyzed carefully. Based on this analysis it was established that the minimal acceptable value corresponds to 6 on a scale of 1 to 10. Figure 12 shows the median of the scores assigned by the users to each version of the system.

The results indicate a favorable evaluation, with scores over the minimal acceptance level. However, analyzing the test results obtained when messages were delivered through the smartphone, we see that in question 2 (related to the understandability of the information delivered by the system) the solution did not pass the minimal acceptance criteria. This was because the messages were not delivered as fluently as expected, since there was a poor communication link between the laptop and the smartphone. Particularly a single peer-to-peer link was used to communicate these devices. That situation was then addressed through the use of the HLMP platform [35].

All participants completed the challenge. Their walking speed was (in average) 0.2 m per second using any version of the system. The scores obtained in the rest of the items were similar for both versions of the system. This could mean that the user did not perceive an advantage in the use of a smartphone, although operatively it represented an important contribution.

### 6.2. Second Evaluation Process

The second evaluation process involved a user population and physical scenario different from the previous one. The system prototype used in this tests was improved, mainly in terms of performance, compared to the one used in the previous experiment. The only change done to the system was the use of HLMP as the communication platform that manages the interactions between the notebook and the smartphone. Such a communication infrastructure is able to keep a quite stable communication throughput among devices participating in the MANET, and it also provides advanced services to deal with the packet loss and micro-disconnection that usually affect to this type of communication. The inclusion of HLMP represented an important improvement to the system performance. Although this improvement was not measured formally, it was clearly identified in the video records of the experimentation process and also in the users' comments.

**Figure 13.**
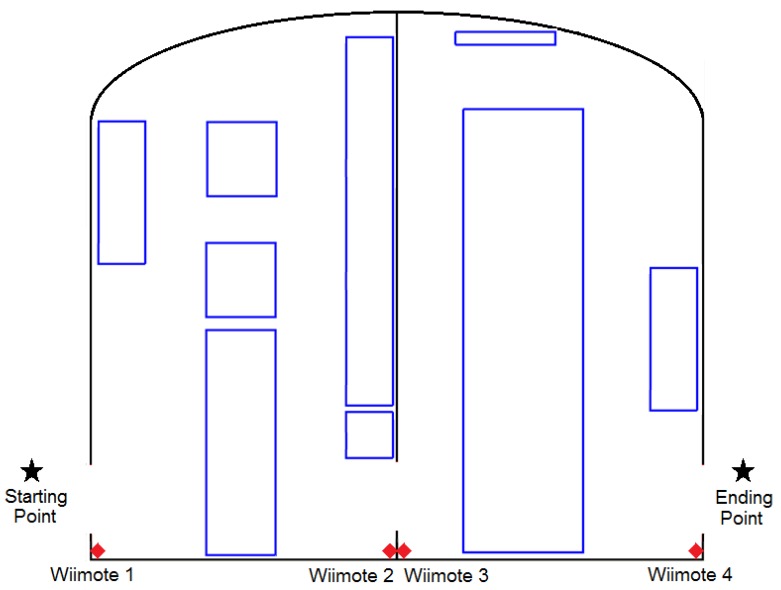
Distribution “A” of the experimentation area.

Nine people participated in this experimentation process: two blind people and the rest were blindfolded engineering students. The physical scenario involved two rooms of 20 m^2^ each (Figure 13). The challenge for the participants was similar to the previous one, but now the experimentation area involved two rooms. In addition, the participants used two settings during the tests: (1) the navigation system with the smartphone, or (2) just their intuition and walking capabilities.

**Figure 14.**
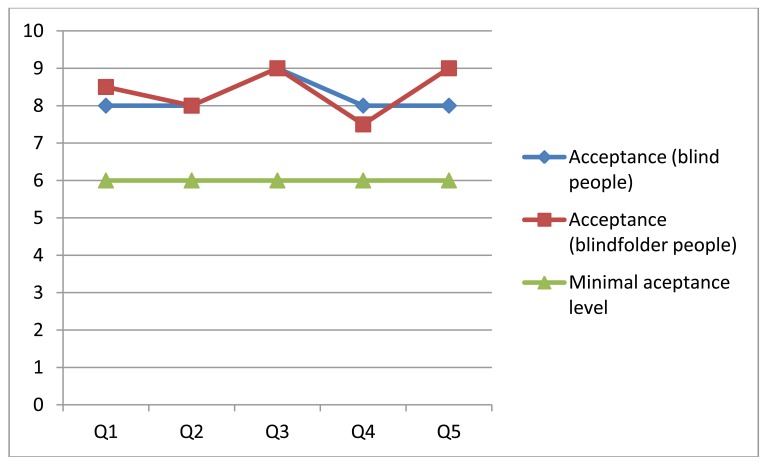
Median of the experimental results for blind and blindfolded people.

After the test the people responded to the same questions as in the previous experience. The obtained results are shown in Figure 14. Although the number of participants is still low, their feeling about the usability and usefulness of the navigation system is good, and their perception is better than the previous experience. A possible reason for that is performance improvement that was done on this version of the navigation system. These results also show almost no difference among the perception of the blind and blindfolded people regarding the navigation system.

Like in previous case, all participants were able to complete the challenge when they used the navigation system. Blind people and just one blindfolded person also completed the challenge without navigation support. Table 1 summarizes the average walking speeds of each test, considering just the completed challenges. Each test was recorded in video, which allowed us then to determine times, distances and walking speeds. These results show that blind and blindfolded people improved their walking speed when the navigation system was used, which indicates the system was useful.

### 6.3. Evaluation of Transversal Issues

During the second evaluation process, some additional metrics were captured to evaluate transversal aspects of the solution. In order to understand the system *performance*, we measured the time period between the instant in which the user pushes the button (asking for navigation support) and the instant in which the first voice message is delivered to the user. Such a period was below 2 s in 90% of the cases. There was no difference if the user was on the move or stationary when asked for supporting information. These preliminary numbers and also the responses to question 4 (shown in the two previous experimentation processes) lead us to believe that the system has an acceptable performance level.

Considering all cases when the white cane was in an area visible by both Wiimotes (see Figure 7), the system was able to deliver *accurate* and *useful* information to the end-user. Considering these numbers and the responses to questions 3 and 5 we can say that the system seems to be useful and accurate enough to support the navigation for blind people in indoor environments.

The system *availability* was also deemed acceptable. The navigation services were available at all times, however the positioning strategy was able to accurately determine the user position when the white cane was in the area visible by the Wiimotes. It represents approximately 65% of the room area (see Figure 7). In areas visible just by one Wiimote (*i.e.*, approximately 25% of the room area) the positioning process was conducted using an estimation based on the last known user's position and the velocity and direction of the user's movement. Ten information requests were conducted in those partially visible areas. In 9 of 10 cases the system was able to provide useful (but not accurate) information to the user.

### 6.4. Discussion

Considering the requirements defined in Section 3 (*user-centric*, *performance*, *usability*, *usefulness* and *economical feasibility*), we can say that the system addresses most of them. Concerning this last requirement, it is clear that the cost of this solution can be reduced considerably if specialized hardware is used, e.g., an infrared camera instead of a Wiimote.

The *availability* of the solution was partially addressed in the current version of the system. Although the system is potentially able to manage large areas, its main limitation is the use of Bluetooth that has a short communication threshold. However this limitation can be overcame just using WiFi communication.

The user *individualization* and the support for *multiple users* were not formally considered in the current version of the system; however they were considered in the navigation model. Due to every room and user have an individual identification, the system can manage collections of these elements by extending the XML file. The only concern could be the system performance when a large number of these components are managed simultaneously by a simple computer. Particularly the network throughput could represent a bottleneck negatively affecting the performance, and therefore the usability and usefulness of the system. This issue can be addressed by distributing the coordination process over more than one computer.

## 7. Conclusions and Further Work

This article presented the prototype of a micro-navigation system that helps the visually impaired to ambulate within indoor environments. The system uses few components and accessible technology. The results of the preliminary tests show that the solution is useful and usable to guide the user in indoor environments. However, it is important to continue testing the solution in real environments, involving visually impaired people to obtain feedback that allows us to improve the proposal in the right direction.

This solution not only allows a user with visual disabilities to ambulate into an indoor environment while avoiding obstacles, but it could also help them interact with the environment, given that the system has mapped all the objects found therein. For example, if the user wants to sit down to rest, the system can tell him where a chair or a sofa is situated. The system can also be used to promote face-to-face meeting of blind people in built areas

Regarding the initial data capture (*i.e.*, initial settings), the article presents a software application that lets the developer create room maps in a graphic way, by drawing the walls and objects. This tool translates this vector map to the XML file required by the system, which simplifies the rooms' specification and the system deployment.

Of course, the most important limitation of the system is that it will fail if a piece of furniture, for instance a chair, is moved. When an object in the environment is moved, the map needs to be refreshed. However, this could be implemented in places that do not change frequently, like museums, theaters, hospitals, public administration offices and buildings halls.

Although the developed prototype and the pre-experimentation phase met all our expectations, more rigorous experiments must be designed and conducted to identify the real strengths and weaknesses of this proposal. Particularly, various non-functional requirements such as privacy, security and interoperability must be formally addressed by this proposal.

The algorithm used for detecting the objects and the movement of the users in the environment works satisfactorily. However, different algorithms could be tested in order to improve the system accuracy.

The limits of this proposal need to be established in order to identify the scenarios where the system can be a contribution for the visually impaired. That study will also help determine improvement areas of this solution.

## Figures and Tables

**Table 1.** Summary of walking speeds.

	**Blind People**	**Blindfolded People**
With Navigation Support	0.7 m/s	0.2 m/s
Without Navigation Support	0.4 m/s	0.05 m/s
